# To the Editors of the Medical and Physical Journal

**Published:** 1803-06-01

**Authors:** G. Rogers

**Affiliations:** Maningtree


### *To the Editors of the Medical and Physical Journal.*

GENTLEMEN,

HAVING lately met with a striking instance of Cow-Pox Inoculation superseding the Small-pox contagion, induces me to avail myself of the first opportunity of communicating it to you, in order that it may be inserted in your valuable monthly publication, if worthy of notice. How far the previous exposure to Small-pox effluvia might have tended to retard the usual progress of the Vaccine disease, in the subsequent case, I will not venture to decide; but surely it affords an excellent proof of the propriety of urging Vaccination to the greatest extent, and not despairing, on similar occasions, to arrest the progress of that dreadful distemper the Small-pox.

John Vice, æt. 14, having been ill four or five days with pain in his head, sickness, and violent pain in his bones, was found partially covered with an eruption on Friday morning, March 18, which was not suspected to be variolous till the following Monday. Being desired to visit him for the first time on this day, I could not hesitate immediately pronouncing him to be labouring under Small-pox, and earnestly recommended his brother to undergo the process of vaccination as speedily as possible, to avoid the contagion. As these boys had been hitherto pretty constantly together, and sleeping in the same bed, there appeared to me no very great prospect of success, and none to the mother, who for some time objected to my proposal: but as the number of children in our neighbourhood not having had the Small-pox was considerable, I thought it a duty incumbent upon me to *endeavour* to cut off this raging disorder in its infant state. Permission being granted, I accordingly inoculated George Vice, æt. 11, on the following Tuesday morning, March 22, with fresh Vaccine virus, and having separated them the preceding day, desired that no further communication should be had between them. On Sunday 27, very little appearance of inflammation on the inoculated part, and scarcely sufficient to believe it had succeeded. On Wednesday 30, the arm still appeared slightly inflamed; but, on Friday, April 1, it began to advance a little. On Saturday and Sunday its advancement was accompanied with the true characteristic symptoms of constitutional affection, and he felt felt exceedingly ill. Monday, April 4, the arm continued considerably inflamed, but the grievous symptoms mitigated, he was able to walk about a little, and began to appear as lively and brisk as usual; and a few days after the dark incrustation formed over the inoculated part, the inflammatory process subsided, and the boy appeared quite well.

I am, &c.

*Maningtree, April* 30, 1803. G. ROGERS.

